# “Where my responsibility lies”: Reflecting on medicine during the Holocaust to support personal and professional identity formation in health professions education

**DOI:** 10.3205/zma001606

**Published:** 2023-04-17

**Authors:** Madelin S. Riesen, Claudia Kiessling, Diethard Tauschel, Hedy S. Wald

**Affiliations:** 1 Witten/Herdecke University, Faculty of Health, Witten, Germany; 2 Witten/Herdecke University, Faculty of Health, Chair for the Education of Personal and Interpersonal Competences in Health Care, Witten, Germany; 3 Witten/Herdecke University, Faculty of Health, Integrated Curriculum for Anthroposophic Medicine (ICURAM) within the Professorship for Education, Training and Continuing Education in Anthroposophic Medicine, Witten, Germany; 4 Warren Alpert Medical School of Brown University, Department of Family Medicine, Lancet Commission on Medicine and the Holocaust, Providence/RI, U.S.A

**Keywords:** professional identity formation, medical education, medicine during the Holocaust

## Abstract

**Objectives:**

Physicians and the medical/scientific establishment during Nazism and the Holocaust committed egregious ethical violations including complicity with genocide. Critical reflection on this history serves as a powerful platform for scaffolding morally resilient professional identity formation (PIF) with striking relevance for contemporary health professions education and practice. Study aim was to explore the impact of an Auschwitz Memorial study trip within the context of a medicine during Nazism and the Holocaust curriculum on students’ personal and PIF.

**Methods:**

The authors analyzed 44 medical and psychology students’ reflective writings from a 2019 Auschwitz Memorial study trip using immersion-crystallization qualitative thematic analysis.

**Results:**

Six distinct themes and 22 subthemes were identified and mapped to a reflective learning process model:

*1. “What am I bringing?” *

*2. “What am I experiencing through the curriculum?” *

*3. “What am I initially becoming aware of as a first response?” *

*4./5. “How and what am I processing?” *

*6. “What am I taking with me?”*

Particularly compelling subthemes of *power of the place, emotional experience, reflection on myself as a moral person,* and *contemporary relevance* referred to impactful course elements.

**Conclusions:**

This curriculum catalyzed a critically reflective learning/meaning-making process supporting personal and PIF including critical consciousness, ethical awareness, and professional values. Formative curriculum elements include narrative, supporting emotional aspects of learning, and guided reflection on moral implications. The authors propose Medicine during Nazism and the Holocaust curriculum as a fundamental health professions education component cultivating attitudes, values, and behaviors for empathic, moral leadership within inevitable healthcare challenges.

## Zusammenfassung

**Zielsetzung:**

Ärzt*innen und medizinische/wissenschaftliche Institutionen haben während des Nationalsozialismus und des Holocaust schwere, ethisch nicht zu vertretende Verbrechen begangen, einschließlich der Mitwirkung am Völkermord. Die kritische Reflexion über diesen Teil der Vergangenheit kann als wirkungsvolle Grundlage für die persönliche und berufliche Identitätsbildung, mit einer intensiven Auseinandersetzung über ethische Werte und Normen dienen. Diese ist für die heutige Ausbildung und Berufsausübung in den Gesundheitsberufen von großer Bedeutung. Ziel der Studie war es, die Effekte einer Exkursion zur Gedenkstätte Auschwitz im Rahmen des Curriculums „Ärztliche Bewusstseinsbildung und Ethik am Beispiel der Medizin im Nationalsozialismus“ auf die persönliche und berufliche Identitätsbildung von Studierenden zu untersuchen.

**Methoden:**

Reflective Writings von 44 Medizin- und Psychologiestudierenden einer Exkursion zur Gedenkstätte Auschwitz im Jahr 2019 wurden mittels thematischer Analyse (Immersion-Kristallisations-Ansatz) qualitativ ausgewertet.

**Ergebnisse:**

Es wurden sechs verschiedene Themen und 22 Unterthemen identifiziert und in ein Modell des reflexiven Lernprozesses gemappt:

*1. „Was bringe ich mit?“ *

*2. „Was erlebe ich durch das Curriculum?“ *

*3. „Was nehme ich initial als erste Reaktion bewusst wahr?“ *

*4./5. „Wie und was verarbeite ich?“ *

*6. „Was nehme ich mit?“ *

Besonders hervorstechende Unterthemen wie *Macht des Ortes, emotionales Erleben, Reflexion über mich selbst als moralische Person* und *aktuelle Relevanz* deuteten auf wirkungsvolle Kurselemente hin.

**Schlussfolgerungen:**

Dieses Curriculum katalysierte einen kritisch reflexiven Lern-/Sinnfindungsprozess, der die persönliche und berufliche Identitätsbildung unterstützt, einschließlich des kritischen Bewusstseins, des ethischen Gewahrseins und der professionellen Werte. Die gestalterischen Elemente des Curriculums beinhalteten Erzählungen (Narrative), die Unterstützung emotionaler Aspekte des Lernens und die angeleitete Reflexion über ethisch-moralische Implikationen. Ein Curriculum „Medizin während des Nationalsozialismus und des Holocaust“ kultiviert Einstellungen, Werte und Verhalten für eine empathische, moralische Orientierung bei den unvermeidlichen Herausforderungen im Gesundheitswesen. Es sollte einen fundamentalen Bestandteil der Ausbildung in den Gesundheitsberufen darstellen.

## Introduction

*“To be a physician requires a transformation of the individual - one does not simply learn to be a physician, one becomes a physician” *[1]*.*

Professional identity formation, (PIF), i.e. “a representation of self, achieved in stages over time during which the characteristics, values, and norms of the medical profession are internalized, resulting in an individual thinking, acting, and feeling like a physician” [2] is a fundamental goal of medical education [3]. Through social interaction with the community of practice, learners move from legitimate peripheral participation to full participation within the community of practice. There is, in essence, a socialization process of negotiation with accepting, compromising or rejecting norms, beliefs and values of medicine’s community of practice [2]. PIF is an active, constructive process [4] with formative educational experiences aiming to “encourage trainees to act as intentional agents in the making of their professional selves.” [5]. PIF linked to personal identity development [4], [6], is entwined with moral development [7] aiming to “anchor students to foundational principles while helping them navigate inevitable moral conflicts in medical practice” [8]. Creating an educational roadmap for this challenges medical educators given moral complexities within medicine and need for a more explicit PIF curriculum [4].

Learning about and critically reflecting on the history of medicine during Nazism and the Holocaust (MDNH) is described as a powerful platform for scaffolding development of morally resilient PIF of a health professional [9], [10] as trainees engage personally and consider implications of this history for their moral agency and for ethics within the profession. Such reflection heightens awareness of dehumanizing elements in modern medicine [11], [12] and ideally “helps equip learners with a moral compass for navigating the future of medical practice and inherent ethical challenges” [10]. This history of medicine within the medical humanities is integral to medical education more generally given calls to transform medical education for development of learners’ capacities beyond clinical competencies (e.g. integration of ethics including ethical vigilance, compassion, humanism) [3], [9]. The 2017 Galilee Declaration [https://bit.ly/3K0GFt5], therefore, called upon medical schools and other healthcare institutions to incorporate teaching the history of MDNH [10] as a distinctive, key, and extreme example of medicine’s failure [13]. A *The Lancet* editorial described this teaching as a “medical imperative” [14] and “instructive” of “incremental steps that can lead to inhumane ethical transgressions in medicine” [15]. The *Lancet Commission on Medicine and the Holocaust* thus proposes “educational approaches using this history to promote ethical conduct, compassionate identity formation, and moral development” [16].

The tragic fact of such egregious health professional involvement in crimes against humanity has affected almost every aspect of modern bioethics [17] with contemporary relevance for issues such as power hierarchies/abuse of power, human subjects research/obtaining valid informed consent, public health policy, influence of economic and political issues on one’s work, dual loyalties, anti-bias, diversity/equity/inclusion, resource allocation, beginning and end of life, and challenges of genomics/technology expansion. While the Nuremberg Code is included in research ethics teaching, low level of medical students’ knowledge on the history of MDNH has been reported internationally [18], [19], [20] and inclusion of this study domain as a medical education requirement is relatively sparse. A national survey on “history, theory and ethics of medicine” compulsory curriculum, for example, reported faculty at 10 of 29 German medical schools endorsing this subject content as necessary within this compulsory curriculum (topic most endorsed of all survey topics), however, it is not required content in all schools [21], [22]. Only 16% of surveyed US and Canadian schools reported required seminars or courses on this topic [17] and only one medical school in Israel (Ben Gurion University) has a required MDNH course [22]. 

While trends of increasing awareness of bioethical issues exist for medical students studying MDNH including visits to actual sites [23], studies of impact of these curricula on personal and PIF are lacking. Our study aim was to explore this within themes of students' reflective writings (RWs) during an Auschwitz Memorial study trip component of a MDNH curriculum and identify curriculum elements supporting personal and PIF in this context.

## Methods

### Setting

Faculty at Witten/Herdecke University (UW/H), Germany, developed a three-year interprofessional curriculum in 2017 in collaboration with the Ita Wegman Institute, Switzerland, entitled “Cultivating Medical Awareness and Ethics through the Example of Medicine in National Socialism” [24]. Students may participate in any or all years of the course, part of the Integrated Curriculum for Anthroposophic Medicine [https://bit.ly/3hgtzeQ] as well as studium fundamentale, an obligatory special study module for all students offering course options to allow “in-depth work and achievement of higher-level competences, such as critical thinking” [25]. Objectives include

1. describing historical events and the role of physicians and the medical establishment, with recognition of development of dehumanizing values, behaviors, and medical systems; the “ethics” of the time,
2. identifying resilience/resistance to such (#1) including one’s own “moral compass” that can be sustained despite social, economic, political, and professional pressures, and
3. demonstrating awareness of contemporary relevance of this history.

The curriculum includes historical content seminars, individual and small group reflection on survivor documentaries, assigned readings (including stories of survival and resistance) [26], [27], and study trips to memorial sites (e.g. Auschwitz Memorial). At the end of each year, students present self-selected topics related to course themes within a service-learning public symposium [24].

In 2019, we aimed for curriculum enrichment with interactive reflective writing (IRW) enhanced reflection to support meaning-making and transformative learning [28]. Benefits of IRW for fostering reflection and supporting personal and PIF are well documented [29], [30], [31], [32]. Study trip curriculum iteration included a health humanities component i.e. IRW, poetry, art, music, as well as meditative practice. Benefits of integrating humanities into medical education [33], [34] include moral sensibility [35], promoting humanism [36], reflection supporting practical wisdom [4], PIF [37], and sense of social responsibility [38].

#### Data collection and data management

Forty-six students (39 medical, 7 psychology) in various training years participated in the 2019 course and four-day study trip (100% of course participants on the trip). Students were invited to voluntary IRW sessions each evening. RWs were introduced by a prompt related to the day’s tour and lecture content including topics of dehumanization in medicine, healers becoming killers, stories of resistance and moral courage, and impact on sense of self, professional identity, and/or future practice within contemporary ethical challenges. Prompts, which also included poetry and pertinent quotations of researchers and writers, served as critical reflection triggers. The structure of the writing prompts allowed flexibility in student response and a “free write” option was included. 

Thirty-four students voluntarily submitted 91 RWs including ten RWs from three psychology students. Students used self-selected identifier codes for the RWs with a maximum of four RWs per student (related to four IRW sessions). Identifier codes were converted into consecutive ID numbers (e.g. 14.3). Various demographics were not collected to maintain anonymity. Forty-four randomly selected RWs (50%) representing 27 students were translated into English by a professional translator to enable all researchers’ participation in the analyses. RWs of author MSR as a curriculum participant were excluded. This study was approved by the Ethics Committee of UW/H. 

#### Data analysis 

The interprofessional, intergenerational research team brought a wide array of expertise to the data analysis, contributing pertinent researcher characteristics and reflexivity to the qualitative assessment [39], [40]. The research team included a sixth-year medical student (MSR), a professor of family medicine/clinical psychologist with IRW, PIF, and qualitative research experience (HSW), and a professor of medical education with research experience in qualitative analysis and in curriculum development, with a dissertation on medicine during National Socialism (CK).

We conducted qualitative thematic analyses [41] of students’ RWs, using the English translation by all researchers. Our ongoing team reflection included addressing how data analysis may be influenced by different cultural and/or familial backgrounds of the researchers, e.g. two German researchers and one U.S. researcher. Any questions regarding cultural or language interpretations of RWs were discussed and the original German language version of RWs was reviewed and/or the translator consulted for further clarification when necessary.

Three authors independently performed data analyses using immersion/crystallization method with reading and rereading of narratives with “cognitive and emotional engagement” yielding “intuitive crystallizations” for identifying and extracting subthemes to saturation [39]. Reflexivity practice and dialogue was ongoing during our data analysis process, including awareness of inherent researcher subjectivity. We discussed how each analysis team member’s underlying biases may affect data interpretation [40]. We analyzed four RWs for themes piloting with discrepancies resolved through discussion. Remaining RWs were analyzed in a sequence of four “batches” of 10-14 RWs and then discussed until thematic saturation was achieved as ascertained by consensus of the research team. This included reflecting on how team dynamics could potentially influence our decision-making process as the researcher team consisted of a medical student and two professors. Reaching consensus while giving voice to the student researcher was essential within the analysis. Our analysis of generating subthemes without “a priori” approach of analytic or thematic preconceptions identified and iteratively refined subthemes. We resolved any lack of agreement via videoconference discussion to achieve consensus on final subthemes. After analysis of the group of 44 narratives, we discussed thematic saturation and consented that it was achieved as no new subthemes were generated in the last sets of RWs.

We worked with the final subthemes in iterative cycles to come to consensus for grouping subthemes into topical themes to be mapped to a reflective learning framework. A physician/junior researcher skilled in curriculum development, reflective group work and IRW served as external auditor and reviewed the entire thematic analysis including themes and subthemes, and reflective learning conceptual framework to support trustworthiness and analysis credibility.

## Results

**Figure 1:**
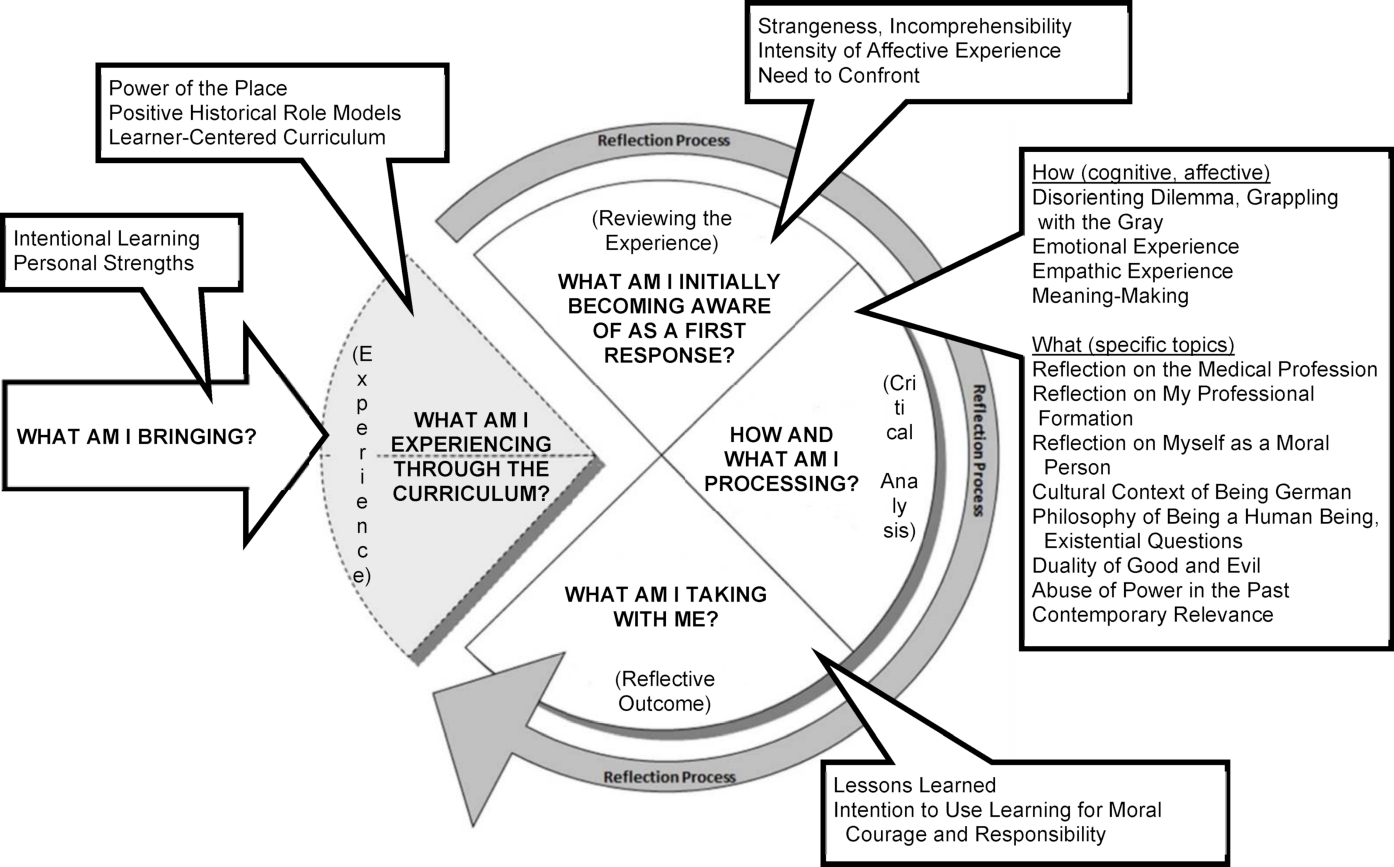
Model of reflective learning process (themes in bold, subthemes are boxed) – adapted from Koole et al. [42]

Six distinct themes of reflective learning with 22 unique subthemes were extracted from the analyses, described below with theme and subtheme titles in italic. Themes, subthemes, and exemplar illustrative quotes are provided in attachment 1 . Themes and subthemes were mapped to a framework of reflective learning process. The iterative process of analyzing and discussing themes and subthemes included discussions about existing reflection models and learning theories to re-evaluate our themes, subthemes and framework of reflective learning process. These were found to be consistent with and extend Koole et al’s [42] reflection model by bringing a student perspective and presented in figure 1 (Fig. 1). 

### Themes

#### Theme 1: “What am I bringing?” 

Reflective learning process begins with what is inherent to the students; what they bring identity- and intention-wise to the experience. Students wrote about *intentional learning* including personal growth and moral development, e.g. “Why did I come here? To strengthen and fine-tune my moral compass.” (11.1). They also wrote about *personal strengths* promoting moral and emotional resilience, e.g. “I would describe my capacity for empathy as strength. […] Faith in love and goodness, coupled with the awareness of the potential evil in us.” (2.3).

##### Theme 2: “What am I experiencing through the curriculum?”

Students reflected on impactful curriculum components, for example, *power of the place*, i.e. being on the actual landscape as significant for contextualizing learning and for emotional engagement: “The need to connect with this place. Physically. Not just to walk on this ground, but to really feel it. Mentally. Absorb, retain and imprint in my brain.” (10.1). Striking specific elements included crematorium remnants with stones placed to memorialize, the infamous “Arbeit macht frei” (“work sets you free”) sign, and victims’ personal belongings, e.g. “It is hard to imagine how many people lost their lives there. And then I saw the unbelievably huge piles of glasses, suitcases, dishes, hair... And it becomes very clear how many people are involved. And still it’s about masses. Masses of people. It’s not hard for me to understand how easy it is to dehumanize.” (6.2).

Students identified and reflected on biographies of *positive historical role models*, including biographies of resistance with role models’ attributes of selflessness, altruism, and humility and the personal relevance, e.g. “I was deeply moved by the story of a prisoner who died after he had taken over the starvation sentence for another prisoner. To show so much selflessness – and in a camp like Auschwitz at that, where hunger, fear and cruelty are constant companions – is very impressive and inspiring. (25.3). 

*Learner-centered program* elements supporting learning emerged including excursion length, group reflective experience, and skilled facilitators as learning catalysts, e.g. “I am overwhelmed with gratitude for Krzysztof’s [program facilitator/educator] words. If only I could bring even a fraction of that love into the world...” (26.3). 

##### Theme 3: “What am I initially becoming aware of as a first response?” 

Awareness at an emotional level and sense of abstraction/absence of cohesive meaning (pre-processing phase) was a first non-cognitive response, i.e. *strangeness and incomprehensibility*: “I don’t get it. I can’t grasp it. I don’t feel prepared. I don’t think there’s anything I can do to be really prepared. […] It is crystal clear and blurred at the same time.” (10.1). Within *intensity of affective experience*, one student wrote: “I try to be generous with myself. […] I cannot force a true understanding. I am awestruck, helpless, empty and full.” (10.1). Some wrote about the Need to Confront the experience to allow connection, e.g. “[…] it was very much abstract, because you have to let the place speak for itself. You have to consider the vast number of victims and perpetrators to feel the impact of the place.” (6.1).

##### Theme 4: “How am I processing? (cognitive, affective)” 

Cognitive and affective elements of reflective learning emerged including experiences of *disorienting dilemma/grappling with the gray* which can be associated with feelings of tension and discomfort, e.g. “Today, there were some situations in which I was forced to question previous perceptions and thoughts: […] biographies […] repeatedly pointed out to me that people have many different motives for their actions. […] I find it difficult to see and weigh up the shades of gray.” (19.2) Also, “The question of how doctors became murderers was also on my mind today.” (9.2) and another brought an evocative image: “[…] the crematorium next to the SS beer bar.” (10.2). 

*Emotional experience*, a reflection element, included a spectrum of often intense emotions such as anger, rage, and shame and at times empathy and love. Challenges in describing feelings and attempts to emotionally calibrate were described: “[…] I’m getting queasy and am overcome by a feeling that I cannot describe at all well - something between disbelief, sadness, anger, despair, fear, helplessness […]. It feels fundamentally wrong, fundamentally evil and insanely absurd, incomprehensible. I can’t understand my feelings either - I’m not properly oscillating with them […] Stunned.” (9.1). Within Empathic Experience, one student wrote: “Today we tried again and again to imagine what it must have been like for people when they arrived at the camp; on the bus, when they passed through the gate and stopped at the selection site.” (11.1). Effortful *meaning-making* was evident, e.g. “The blocks: They are rooms. Bad things have happened there. Yes! But it’s just rooms. I fill them with meaning.” (4.2).

##### Theme 5: “What am I processing? (specific topics)” 

Students critically reflected on specific topics of personal and PIF, philosophical issues, and abuse of power. *Reflection on the medical profession* included awareness of vulnerabilities within the profession such as dehumanization. Questions emerged about “[…] how doctors could come to such a medical ethos […]” (9.4) and further, what constitutes the medical profession’s norms and values including research practice and objectivity: “When do we stop seeing people as human beings? The moment I have to make a diagnosis? The moment when I can no longer open a room for the people seeking help who sit opposite me, because I have to be with myself and go through the criteria in my mind: Sleep problems √ Weight fluctuation √ Persistent depressed mood √ → depression.” (11.2). Within *reflection on my professional formation*, students grappled with implications of atrocities and resistance in this historical context for their own PIF, e.g. “I have found out for myself that it is difficult to dehumanize as long as one approaches the other person individually and understands them as a whole person. I have also understood how much strength is derived from treating people “humanely.” I am convinced that it will be difficult to be a doctor if you forget all this” (6.4) and with implications for a sense of agency, i.e. “What impulse do I take with me:

- becoming more politically *active*;
- more courage to determine my own medical career path;
- actively speaking up and showing commitment in medically and morally precarious situations” (8.4).

*Reflection on myself as a moral person* included reflecting on one’s own moral integrity and ethical conduct and need for intentional moral courage, resilience, and steadfastness, e.g. “recognizing and accepting one’s own shadows, in order to decide against them intentionally + consciously. Inside me, too, is a perpetrator. Do I have the courage to name him and to resist?” (5.4). The *cultural context of being German* emerged, e.g. “The questions why and how always came up. I asked myself if I could understand the cruel actions or if I have to understand them. As a German, do I have to understand what was going on in order to ask for forgiveness in my mind and to express my incredible sadness to the victims? Do I have any responsibility? (as a German?)” (28.2).

Within the *philosophy of being a human being and existential questions* subtheme, students questioned the essential constitution of humankind, fundamentals of morality and origin/nature of a “shift” to immorality, nature of moral responsibility and free will, e.g. “to what extent is every character trait of a person only a result of all their experiences? How much do moral decisions depend on a “random generator”? […] But what does truth mean?” (19.2). The subtheme of *duality of good and evil* included complexities such as existence of morality/immorality and the challenge of comprehending and/or tolerating this tension and complexity, e.g. “complete inability to understand how leading SS doctors and other SS officers could carry out the most horrible atrocities against humans on a daily basis with such perversity and deliberation and, at the same time, had a family of their own, probably loved their wives/husbands and children more than anything.” (12.2). Students contemplated *abuse of power in the past*, e.g. “The stories that impressed me most were those about power - and who uses it for what. I find it “remarkable,” in a way, how one and the same emotion causes an individual to send thousands to their death without batting an eyelid, while at the same time, it caused people to put their life at risk again and again every day, in order to act in a deeply human way, both in the medical and the personal sphere.” (1.3). *Contemporary relevance* of this history emerged, e.g. dehumanization, power hierarchies, healthcare equity, and racism specifically within the clinical context, e.g. “My focus was on the subtle facets of dehumanization in society and medicine, the everyday ones. […] And yet it touches me so much precisely because I am gradually becoming more aware of how racist our society, and of course (pro-hierarchical) institutions like hospitals are. Once you start to pay attention to this (and here your own learned thought patterns are paramount!), it is everywhere.” (26.1).

##### Theme 6: “What am I taking with me?”

*Lessons learned* included the importance of constant self-reflection, discussing feelings and ethical dilemmas, speaking up, being courageous, and being aware of learning from history to inform future action, e.g. “The past is not just a past that should/may be forgotten but […] it carries us further until we become aware of it, want to understand it and set out to understand it and to transform this newly learned awareness into future awareness.” (20.4). Also “I have also understood that it is good to stop and question your work again and again, as well as the system in which you operate.” (6.4). 

*Intention to use learning for moral courage and responsibility* was conveyed with conviction and inspiration, i.e. applying one’s inner moral compass within personal and professional relevance of this history: “I want to initiate change […]. I want to encourage others to do the same. I would like to draw creativity and strength from this tragedy I have experienced in order to be able to change myself and to allow change.” (19.4). Also, “I hope that I have sharpened my senses for dehumanization and that I will find the courage to raise my voice in such situations.” (28.4). 

## Discussion

Our study of health professions students’ RWs during an Auschwitz Memorial study trip component of a MDNH course investigated the impact of such a curriculum on students’ personal and PIF. To our knowledge, this is the first comprehensive qualitative study of outcomes of such a curriculum for health professions students. The curriculum catalyzed a critically reflective learning process [43] including historical knowledge as well as formative experience of grappling with what has been termed as “disorienting dilemmas” [28] such as healers becoming killers, physician vulnerability and fallibility [44], “good/evil” dualities, and challenges of moral courage [45]. Processing within RW for meaning-making supports transformative learning for personal and PIF (generated subthemes), informing attitudes, values, and ideally behaviors for empathic, morally responsible practice. Threshold concepts which include “troublesome knowledge” can and did emerge, defining potentially powerful transformative points in students’ learning experience [45], which may be an enduring imprint. Our findings demonstrate reflective learning on this fundamental topic for scaffolding development of humanistic, morally committed resilient personal and PIF for health professionals [9], [10].

Particularly compelling subthemes (number/nature of quotes) of *power of the place, emotional experience, reflection on myself as a moral person*, and *contemporary relevance* referred to impactful course elements. *Power of the place* or “pedagogy of place” subtheme [46] aligns with descriptions of visits to Holocaust-related sites holding special meaning for medical students [47]. In addition, a majority of RWs included the subtheme of *emotional experience* with strong emotional engagement often noted and which both influences cognitive and motivational processes crucial for learning [48] and is closely related to identity development [49]. This finding aligns with the “link between students' emotions and their identity development in the powerful world of becoming and being a doctor” ([49], p. 174) and discourse on this as potentially contributing to shaping reflective, responsible professional action within “caring identities” ([49], p. 174). Emotional experience can include feeling unsettled within new awareness and “reconstituting” within threshold concepts, i.e. integrating new knowledge into one’s self-concept. Reflection on affect can contribute to the emotional development and wellbeing of a medical student within PIF [30] given that there is an emotional process to becoming a physician [50].

*Reflection on myself as a moral person* highlighted reflection on personal development (which tends to be underemphasized in medical education) in addition to professional development. Reflective process, according to Kegan, spurs developmental move in identity [51]. *Contemporary relevance* was a key thematic outcome with connecting this history to current issues of potential abuse of power and dehumanization, as well as to future ethical dilemmas and challenges in medical education and practice, having moral courage for “speaking up,” respecting diversity, and preserving human dignity. Concerns have been raised about “lack of evolution” of moral reasoning in medical education [52]. Our study outcomes suggested that both content (historical) and process (reflection on action [53] and on being [51], [54]) contribute to development of critical consciousness within active, constructive PIF. Within this curriculum as an “identity enriching” experience [55], we were struck by existential questions emerging within the subtheme of* philosophy of being a human being/existential questions* and the *grappling with the gray* of difficult dualities in an effort to reach a more nuanced understanding. Our study also revealed students’ ongoing reflection with the theme *“What am I taking with me?”* including unanswered questions (in addition to *lessons learned*), a valuable process within lifelong learning and sustaining critical consciousness, resonating with “living the questions” [56], the “right questions.” [57]. According to Kleinman, “we must interrogate our moral life” [35].

IRW can “offer emerging physicians opportunities to more fully incorporate their experiences into a professional identity that embodies reflection, critical awareness, cultural humility, and empathy” [58] as well as critical consciousness and ethical vigilance [9]. The thematic analysis revealed students' reflection supporting the PIF process through both reflection on and vigilance for one’s own moral agency as well as reflection on medicine as a profession and a healthcare system and changes that may be needed in both realms such as equitable access to health care and addressing structural discrimination. This was seen in various subthemes including *reflection on the medical profession, reflection on my professional formation* and *contemporary relevance*.

In general, our theme/subtheme results support an individualized and socialized PIF process for becoming a member of a community of practice, extending the negotiation process [2] with including PIF as being influenced by the history of the profession as well as clinical and non-clinical experiences. Critical reflection on MDNH history triggers PIF processes when presented within powerful course elements such as biographical narratives including historical moral role models, reflection sessions with skilled facilitators, and *power of the place*.

We reviewed various published reflection models [59], [60], [61], [62], [63] as we mapped themes and subthemes to a framework of reflective learning process and found good alignment with the “eclectic” reflection model of Koole and colleagues ([42], p. 2) which includes elements of several existing reflection models. The addition of the theme *“What am I bringing?”* to the Koole et al. [42] reflection model acknowledges students bringing their perspectives (biases?), assumptions, characteristics, and identity(ies) to the learning process, thus encouraging a supportive individualized as well as group approach to teaching, especially within potentially challenging topic content. 

Several students’ drawings and poetry emerged organically during reflective exercises. Biographies of victims/resisters were identified as a key, vivid educational component eliciting empathy and emotional resonance and personal narratives of faculty (including author HSW, a daughter of a survivor) were also cited as impactful.

Limitations of our study include the question of transferability given that the data was collected from one institution in one country (Germany) and exclusively describes the experiences of students who engaged in voluntary RW during a study trip component. Variables including level of training, gender, and/or cultural background are of interest. While future studies may elucidate relative contribution of trip components to this MDNH curriculum, our study revealed significant impact on and pedagogic value for health professions students having this MDNH educational opportunity including a trip component. While foundational PIF concepts have interprofessional relevance, such as “sense of connection to values of the profession” in psychology [64] (and in medicine), curriculum impact on psychology profession-specific PIF may be addressed in the future. As this is an elective course with voluntary participation, we would be interested to study outcomes of a compulsory course and study trip, given the call for such curriculum inclusion in medical education [10]. Data is emerging on positive impact within required seminars and courses [65]. We encourage inclusion of reflective opportunities within such curricula with trained facilitators [65] to promote student engagement and deeper understanding of how this history scaffolds PIF. It would be of interest in future studies to explore nuances of relation to the past and meaning-making within study of this history among students with different cultural backgrounds and what may be shared or differ. While analyses revealed some German culture-specific aspects of the experience, universal themes and subthemes emerged, thus supporting this intervention as a transferable model for teaching in other settings. Key course features, based on our experience, are provided in table 1 (Tab. 1) for facilitating transferability to other learning institutions. Examples of curriculum implementation by various medical schools including grant and foundation supported visits to Holocaust memorial sites/Nazi death camps are available [24], [47], [66] with some positive impact trends [47] and relevance for recent increased Germany medical education curriculum emphasis on PIF [https://gesellschaft-medizinische-ausbildung.org/ausschuesse/professional-identity-formation.html].

The vision of a curriculum with

1. core principles for health professional formation and
2. local/situated relevance with positive role models in one’s specific national context can thus be achieved [67] (see table 1 (Tab. 1)).

Impact of MDNH education on PIF process of negotiating socialized and internalized personal values [7] as well as within the professional developmental lifecycle of graduate and continuing health professions education is of interest [67]. Our study outcomes resonate with recent findings of impact of this history for PIF in nursing education [65], [68]. Ideally, this education can serve as a “practical anchor point, a tool for reflection” [69] triggered by challenges in contemporary medicine and within global citizenship. 

Medical education needs to include studying how the meaning of being a physician and responsibility within the physician-patient relationship changed so thoroughly and radically in Nazi Germany, being distorted to caring for the Volk (state) vs the individual patient. Learners need to grapple with the fundamental changes in professional ethical standards within the symbiosis of medicine and Nazi state policy and recognize how the Holocaust was unlike any other genocide as “the Holocaust occurred with the intellectual support and involvement of the medical and scientific establishment” ([70], p.63). Antisemitism was legitimized within the medical profession. Therefore, even beyond PIF, MDNH is a topic that should be integrated as a mandatory subject in every medical degree program. 

The study of MDNH is compelling for learners at all stages of the professional lifecycle. It serves as an enduring reference point to the antithesis of the qualities we wish to cultivate in a health professional as well as to admirable qualities which would ideally be inspired by examples of resistance and moral courage. We plan to study longitudinal impact of this course on lasting changes in attitudes, values, and behavior as a guiding moral compass within personal transformation and PIF for respecting medicine’s “covenant” [71] with patients and bringing trustworthiness.

## Conclusion

In conclusion, health professions students’ grappling with ethical implications of pervasive physician complicity during Nazism and the Holocaust with extreme perversion of professional ethics [72] as well as examples of resistance/moral courage yielded formative impact. This included sensitized moral compass, heightened awareness of contemporary relevance for preventing abuse of power and respecting diversity, and moral intention to apply their learning responsibly within the profession and society, in line with the need for “socially accountable” health professions education [73]. Compelling RW evidence of such curriculum supporting students’ personal and PIF for humanistic healthcare and health policies lends support to the call for integration of critical reflection on the history of MDNH as “imperative” within all health professions education [10], [14], [72].

*… “Ah, this is where my responsibility lies - never let something like this happen again.” (28.2)*

## Declarations

### Ethics approval

This study was approved by the Ethics Committee of Witten/Herdecke with participants informed that voluntary submission of their anonymous reflective writings would be used in this study. 

#### Authors' contributions

DT was director of the curriculum program. HSW developed and facilitated interactive reflective writing activities. CK, MSR, and HSW analyzed the data. All authors contributed to the manuscript planning and writing.

MSR is a sixth year medical student at Witten/Herdecke University. 

## Acknowledgements

The authors wish to thank Prof. Dr. med. Peter Selg, Ita Wegman Institute for Basic Research into Anthroposophy, Switzerland and Dr. phil. Krzysztof Antonczyk, Auschwitz-Birkenau Memorial and Museum, Poland for facilitating the study trip and educational presentations, Clarissa Frehle, Witten/Herdecke University, Faculty of Health, Germany, for analysis review, and Jeffrey M. Borkan, MD, PhD, Warren Alpert Medical School of Brown University, RI, USA for helpful feedback on methodology.

## Competing interests

The authors declare that they have no competing interests. 

## Supplementary Material

Thematic content of students’ reflective writings with illustrative quotes and reflective learning process themes and subthemes

## Figures and Tables

**Table 1:**
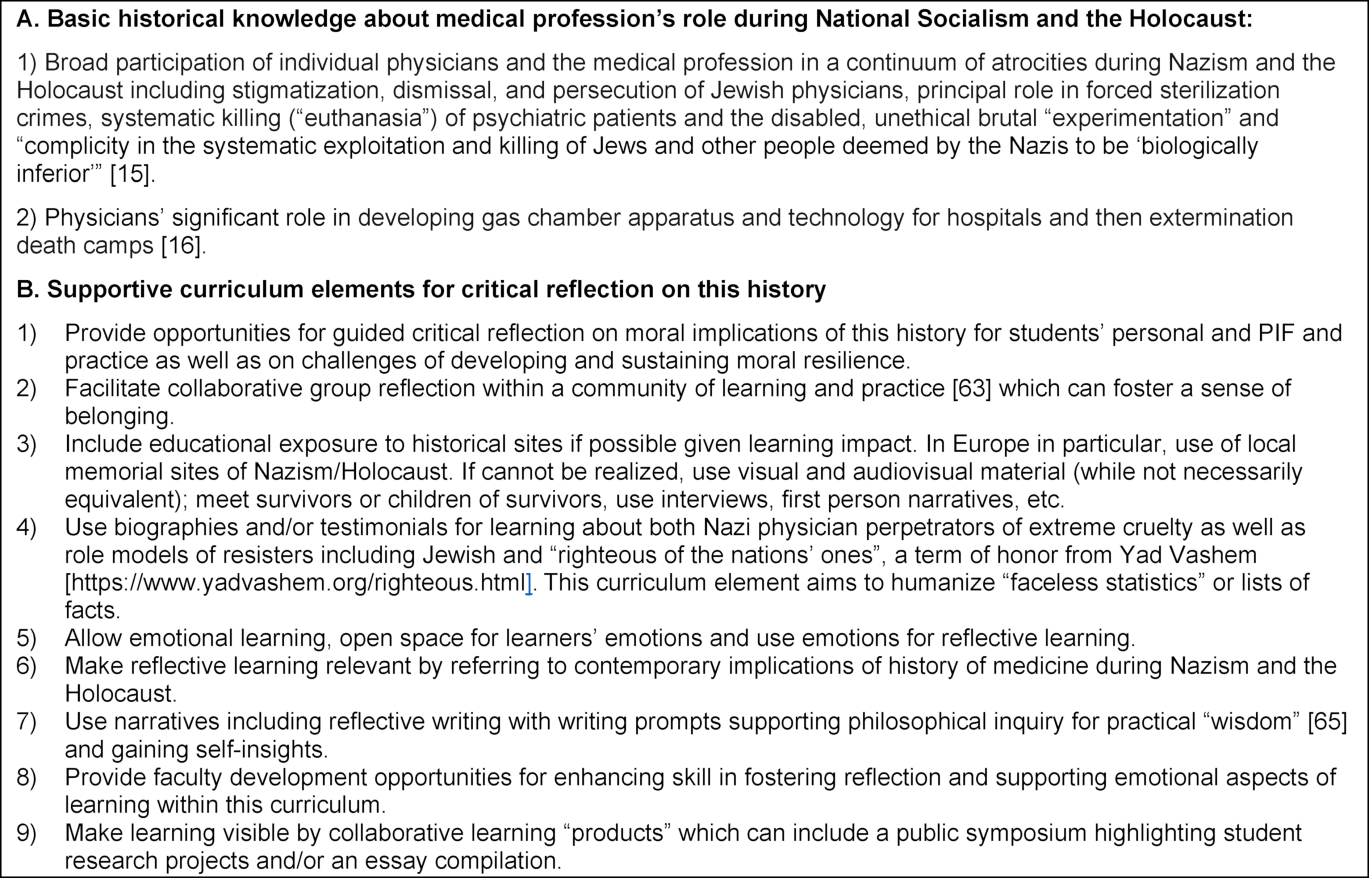
Key features for a “Holocaust and Medicine” curriculum
