# Supplementary material for: “Where my responsibility lies”: Reflecting on medicine during the Holocaust to support personal and professional identity formation in health professions education
Source: GMS J Med Educ. 2023 Apr 17;40(2):Doc24. doi: 10.3205/zma001606 (PMC10285371; doi:10.3205/zma001606)
Supplement: Thematic content of students’ reflective writings with illustrative quotes and reflective learning process themes and subthemes [file JME-40-24-s-001.pdf]

Attachment 1: Thematic content of students' reflective writings with illustrative quotes and reflective learning process themes and subthemes

Subthemes	Quotes
**<u>Theme 1:</u><br><u>"What am I Bringing?"</u>**	
**Intentional Learning**	*I have come here because I wanted to understand whether man per se carries an "evil" part in himself or not and to what extent the phenomenon of the "Final Solution" can - or cannot - be explained by it... (8.1)*<br>*Today, the first day, is enough to show me why I came here. I want myself to know what happened here! I want to have a picture, stories, biographies and names. I have to be here, for the murdered people and for myself. (16.1)*
**Personal Strengths**	*I have my heart-strength within me. The light that is always there, that can fill me and shine into the world. The love that can transform everything. And I have the determination to continue to nurture, strengthen and expand this inner quality. (7.3)*
**<u>Theme 2:</u><br><u>"What am I Experiencing through the Curriculum?"</u>**	
**Power of the Place**	*A) Power of the place*<br>*[…] you have to let the place speak for itself. (6.1)*<br>*I was impressed to learn things about myself and my values in a place with such a terrible history. It was a place where I got to know courage in a very different way. (28.4)*<br><br>*B) Specific elements that struck students*<br>*Block 10 was an extreme case; the building was quiet […]. (6.2) [authors' note: Block 10 was site of brutal gynecological experimentation on women]*<br>*Today I was surprised how quiet and seemingly peaceful the place is. The only element left that is still imbued with agony and despair are the electric fences. (8.1)*<br>*The stones that people have recently laid on the edge of the crematorium connect what was then with what is today. This is a mass grave. People commemorate here. If one truly understood what happened here, it would be unbearable. (10.1)*<br>*Impressive is that resisting always seems possible. It begins with the "B" of the sign at the entrance of the main camp being turned upside down and continues throughout the entire camp life. (25.4)*
**Positive Historical Role Models**	*Today I was able to strongly identify with the story of the doctor, who is said to have had a warm, light-filled gaze while distributing the medication among the sick. When she sat down with the dying, her eyes reflected the pain. (2.3)*<br>*As I walked across the concentration camp grounds and thought of the strong men and women of the resistance […] in this overwhelming setting I was suddenly no longer so sure whether I would have withstood the pressure, whether I would have been so selfless, whether I would have exposed wrongdoings, whether I would have looked closely, whether I would have acted right. (6.1)*<br>*Resistance was possible even in the most senseless and hopeless situation […]. (15.4)*<br>*I was very moved when I understood that the "victims" of Auschwitz were not only victims, but also understood their own heroic strength. I also understood that I should consider them less in terms of the masses of people and more of individual destinies. (6.4)*<br>*I think that Hautval's ability to forget herself left more space for her lack of vanity and her empathy. I would like to work on this myself in my everyday life. To develop the ability to forget myself, to grant my highly developed empathy a space that is free of my ego. (22.3) [authors' note: Adélaïde Hautval was a French psychiatrist who was a resister while imprisoned in Auschwitz]*

**Learner-Centered Program Elements**	*Length of excursion*<br>• *After one day I still don't feel that I have accepted all these impressions fully and I am therefore glad that the course gives me the opportunity to spend several days studying this topic in depth. (24.1).*<br>*Being in Auschwitz as a group*<br>• *It felt completely different to walk through the camp alone, in pairs, on our own, than in the morning, when we walked as a group. (10.2)*<br>• *We are going on a journey together with a nice group of people in beautiful early summer weather - it smells of freshly cut grass, of smoke - like on the way to a barbecue on green meadows. Nature awakens to spring & we enjoy the warm sun & being together - until suddenly I realize through which gate we are walking and which walls surround us. (9.1)*<br>*Space for exchange*<br>• *It was here that it became clear to me how important it is to discuss feelings and ethical behavior, to rethink them over and over again and to get into an exchange. (28.4)*<br>*Facilitators as catalysts*<br>• *Experiencing Peter Selg and Krzysztof [as people] has impressed me: they are interested in the matter at hand, very differentiated, clear, examining layers, personal stories. (15.4)*<br>• *The moment Hedy showed these pictures, I realized how chaotic, confused I felt. (13.3)*
**Theme 3:<br>"What am I Initially Becoming Aware of as a First Response?"**	
**Strangeness, Incomprehensibility**	*It's overwhelming to be at the scene of such a crime. […] But even when you abstract the place and look at the factorylike site in its new form, you can feel its strangeness. (6.1)*<br>*At first, when I saw the fences and the barracks from the bus, it was absolutely absurd to be here now. Almost as if I had to tell myself over and over again what happened here, as I couldn't really understand it. (16.1)*
**Intensity of Affective Experience**	*Right now I feel incredibly vulnerable. I can't tell exactly where it's coming from. […] The unbelievable horror, the absurdity of the system, the energy, the planning, the effort leave me stunned. (13.3)*
**Need to Confront**	*Today we have all found a way of dealing with Auschwitz. It's like communication, you can't not communicate. You can't not deal with Auschwitz either. (10.2)*
**Theme 4:<br>"How am I Processing? (cognitive, affective)**	
**Disorienting Dilemma, Grappling with the Gray**	*This is the only way how I can become aware of the complexity of the subject matter. I have thought a lot about images of man and what drives man to act. I believe that there is something evil in every person. This is not inhuman. But in every human being there is also a good light. All that's left to do now is find out what emerges and when. (6.4)*<br>*After today & following some other experiences, I think that probably (almost) every person can be demoralized - if you do it step by step & constantly. It goes like this: first small transgression that are considered "immoral" but not yet "bad," but which are then "positively reinforced," e.g. played down, praised etc. And since this "small" demoralization is now the "measure of all things," another small transgression can be demanded in the next step and it can once again be "justified." I can imagine that in this way, by "slowly hollowing out the stone," awareness can get lost or at least be greatly diminished. What was "normal" changes. Soon other things will be "normal," simply due to familiarization, rather than due to a conscious shift. I think this is very dangerous. Because it can happen seemingly unnoticed. (9.2)*

Attachment 1 to Riesen MS, Kiessling C, Tauschel D, Wald HS. *"Where my responsibility lies": Reflecting on medicine during the Holocaust to support personal and professional indentity formation in health professions educations.* GMS J Med Educ. 2023;40(2):Doc24. DOI: 10.3205/zma001605

**Emotional Experience**	*Anger. Anger at all that madness. (4.1)*<br>*Because I carry a rage inside me about what has happened […]. (19.2)*<br>*[…] I felt very small and ashamed […]. (6.1)*<br>*A gate with the inscription "Arbeit macht frei" surrounded by many electric fences. A feeling of tension and anxiety comes over me - what will the buildings hold? (8.2) [authors' note: infamous "Arbeit macht frei" sign translated as "work sets you free"]*<br>*I would like to accept Krzysztof's gift and express my feelings in pictures: Inside me is a tank full of empathy, tolerance and love. This tank is drained daily and drop by drop, because human compassion is often a tour de force. Krzysztof has filled this tank to the brim with his story (and not only him) and, despite all the horror & fear, has strengthened me and made me feel hopeful. This is how I will leave this place and remember it for a long time. (27.4)*
**Empathic Experience**	*I almost required myself to be able to feel everything here, everything, the realities, the hunger, the absolute suffering, the inhumanity. But it was not like that, it is the individual stories and biographies that I am able to feel. I stand in the locations and try to imagine what they must have looked like back then, it is unimaginable to me. (16.1)*
**Meaning-Making**	*The more is revealed, the more complex and chaotic everything seems... (8.1)*<br>*The responsibility, not just with regard to knowledge, but also the responsibility that has to bear consequences for today's political events. While I already brought these convictions to Auschwitz with me, today they suddenly became palpable on a deep emotional level, as if I had said them before but never really understood them. (26.3)*
**Theme 5: "What am I Processing? (specific topics)**	
**Reflection on the Medical Profession**	*After today & following some other experiences, I think that probably (almost) every person can be demoralized - if you do it step by step & constantly. (9.2)*<br>*One concrete thing that has occupied me very much is […] that it became "okay & ethically correct" to select people based on their "race," genetics & origin/community of faith, to declare them unlivable & kill them. […] Likewise, it has become important to me once again to look at the objectivity, which is so highly held in medicine, critically & carefully & to cultivate a conscious handling of it, in order not to let too much distance & supposed objectivity (which can quickly slip into "materialism" & functionalism of humans) get into my medical doing. (9.4)*<br>*It is claimed that the healthcare system is about health. But it's money that plays the central role. And power. (4.1)*
**Reflection on My Professional Formation**	*The experience with the active - small and large - resistance, the saying no, has kept me very busy. I would like to strengthen, sensitively perceive and develop this in my private and professional life. So also in professional-political, as well as ethically difficult medical (everyday) situations, I want to let my ethical and moral voices speak more & stand by them & give them a voice, instead of letting them remain silent due to hierarchical structures & insecurity & letting things happen behind which I cannot stand well. (9.4)*<br>*What do these questions mean for my later cooperation in a team and a place of diversity, like a hospital? (2.4)*
**Reflection on Myself as a Moral Person**	*And if someone follows blindly. But I do it often, too. And I don't fight back. I don't question. Wake up. Now! (4.2)*<br>*What I found most impressive were the seemingly inexhaustible resources of humanity, courage and willpower that people showed in this place, in the face of total destruction of those very qualities. And therefore the question opens up for me and my personal path, how I can invest these resources within me. (1.4)*<br>*So you don't even have to "justify yourself" to yourself, because you don't even have to really notice how the needle of the moral compass is constantly shifting by degrees. Questions to myself: How can I make sure that I don't undergo such a "brainwashing" (externally or internally)? Can I find structures/methods/possibilities that help me to reflect myself at regular intervals & to question whether I am still on the morally "right track"? (9.2)*<br>*How much monster is inside me? (12.2)*

**Cultural Context of Being German**	*I was impressed by those who resisted, rebelled, even though they had to expect painful consequences. I was impressed by Krzysztof […], who gave us his love and affection, although he had every right to hate us as Germans. (27.4)*<br>*It was terrible to see the pictures. Especially the corridor with the portraits of the prisoners. It was incredibly oppressive. Many had a rigid facial expression, I felt so accused, it was oppressive in every look. (6.2)*
**Philosophy of Being a Human Being, Existential Questions**	*How can you get a person to be as fundamentally unempathetic as it was necessary for SS officers & doctors to be to do such things? Is it possible to develop such a hostile attitude in every person? Or does it require extreme experiences or very early imprinting in an immoral direction? (9.2)*<br>*So where does this disease start? Where are the first signs of symptoms? Doesn't every type, every attempt, every intention of dehumanization, abuse, persecution of another person already set the course towards this "end-stage disease"? Isn't the inner shift that such a person performs in this situation strongly related/similar to the inner shift of the SS/Nazi criminals of that time? (7.2)*
**Duality of Good and Evil**	*We let our gaze glide over the expanse of the grounds. It is warm and grey sunlight stands out against the pigeon blue clouds. A battle between light and dark, as it takes place in every single human being. Here the dark side has won, here the hearts of the people turned black. And what about the hearts of the victims? What does this fight mean for us humans today? (11.1)*<br>*Faith in love and goodness, coupled with the awareness of the potential evil in us. (2.3)*<br>*Considering this experience and the stories I have heard - can I divide my world and my view of history into good and evil? Wouldn't I have to look at the exact background and motivations of each individual player before making a judgement? (19.2)*<br>*Does everyone carry the potential for "good" & "evil" in themselves? And different forms of that good and evil develop depending on what is promoted & used? (9.2)*
**Abuse of Power in the Past**	*It was hard to imagine how hundreds of women were used and killed there as test objects. I wonder how a doctor can stand to act like that. (6.2)*<br>*Doctors became murderers for 2 reasons: either they were psychopaths or they never questioned or reflected the ideologies + the racial theories they were taught. - Nobody should decide about life & death of another. (28.2)*<br>*Was there really a conviction behind it to do something meaningful? Or to do the right thing? Or did they distance themselves fully? Fear? Power? Greed? (9.1)*
**Contemporary Relevance**	*I remember many situations in which unconscious, narcotized patients were mocked […] Enjoying & abusing one's own (superior) power with defenseless patients. (9.1)*<br>*Power plays an important role. Dominance. Aggression! Power also plays a central role in hospitals and the healthcare system, power over life. Deciding over the lives of others. Being superior to others. […] Those who have power might also behave irrationally and antisocially. And the more power you have, the more irrationally and insanely you can behave. There are stories of senior consultants who scream, spit and operate without any real indication. Those who have enough power and do not question […] themselves. Power also has something to do with fear! (4.1)*<br>*I know situations in which I would have liked to live out my convictions and attitude more, but I would have had to speak out against a system or against "superiors." I would have risked ending up in an argument or in a worse relationship with the persons concerned. And so I often remain diplomatic. The stories of resistance have shown me that it is important to be courageous and to fight for one's convictions. Today, I am in the happy situation that, in doing so, I do not have to risk my life. (12.3)*<br>*In psychiatric wards; psychiatric hospitals that work with mindfulness-based methods, studies are conducted today and effects are examined on patients. Today. Voluntarily and with informed consent. But how well founded is this "informed consent"? (11.2)*<br>*I believe, once again, that nurturing & maintaining empathy is a great key to humanity. I think that if there is enough empathy for all people (no matter what "group") in a person, it can be prevented that inhumanity creeps in to a great extent. (9.4)*

**Theme 6:<br>"What am I Taking with Me?"**	
**Lessons Learned**	*I was surprised how many positive things you can take away from this place. It is not only a place of destruction and misery, but can also serve as a foundation for new and better things. (25.4)*<br>*It always helps to believe in the good in oneself and to stay true to oneself. (28.4)*<br>*For me, this impulse, this experience has existential significance, when it is understood as an invitation to act consciously and conscientiously, to strive to realize the Good. […] to read, reflect, listen and develop a fundamentally human attitude, which remains steadfast in crises, remains critical, is always attuned towards people, especially towards the other person, and thus ultimately remains honest with itself and the chosen profession/life path […]. (1.4)*<br>*What has changed me is the experience of how close all this happened. It happened here, next door, by and with people like me and you. (18.4)*<br>*Before this day, I was rather convinced resistance must have change a situation with immediate effect. But I have strong memories of the artist who designed the sign "Arbeit macht frei." It had no immediate effect on the situation, but for me it now makes this place not a place that was under the brutal & inhuman control of the Nazis, but a place that shows the identities & resistance of the prisoners as soon as you enter it. In spite of the dictatorship, it becomes obvious on entering the place that something went wrong here: see the "B" [authors' note: sign made with an upside down "B"]. This does not change the fact that millions of people died. But now they are not mere victims, but resistance fighters. With identity & expression. (23.3) [authors' note: infamous "Arbeit macht frei" sign translated as "work sets you free"]*
**Intention to Use Learning for Moral Courage and Responsibility**	<u>Transformation</u>*: 1) My great-grandparents were murdered in a concentration camp. I don't know where, when and how. Paradoxically, my uncles are historians and yet I have never investigated. That is what I will do now. (27.4)*<br>*I wish - stronger in this moment than ever before - to find and use this power for change. There are so many things in medicine and in everyday hospital life that disturb me, paralyze me, drain me of strength - shouldn't I bundle up this resentment and turn it into courage? (19.4)*<br>*I want to remember this feeling later whenever I encounter dehumanization. (6.1)*<br>*A circle of thoughts about questions that cannot be solved, and I have to learn to live without the answer and never let anything like this happen again […]. (28.2)*<br>*If so, then from now on I want to use all my strength and courage in such situations (be it in hospital/therapeutic context, in everyday life or elsewhere - through others or even through myself!) to counter this shift unflinchingly. Against the horror of then and now and for love and goodness in people today and tomorrow! (7.2)*
